# Tolerable amounts of amino acids for human supplementation: summary and lessons from published peer-reviewed studies

**DOI:** 10.1007/s00726-021-03054-z

**Published:** 2021-08-02

**Authors:** François Blachier, Anne Blais, Rajavel Elango, Kuniaki Saito, Yoshiharu Shimomura, Motoni Kadowaki, Hideki Matsumoto

**Affiliations:** 1 Université Paris-Saclay, AgroParisTech, INRAE, UMR PNCA, Paris, France; 2 Department of Pediatrics, School of Population and Public Health, British Columbia Children’s Hospital Research Institute, British Columbia Children’s Hospital, University of British Columbia, Vancouver, Canada; 3 School of Medical Sciences, Fujita Health University, Toyoake, Aichi Japan; 4 College of Bioscience and Biotechnology, Chubu University, Kasugai, Aichi Japan; 5 Niigata Institute of Technology, Niigata, Japan; 6 International Council on Amino Acid Science of Japan, Tokyo, Japan

**Keywords:** Amino acids, Supplementation, Tolerable upper intake level, Rat model, Humans

## Abstract

Amino acid supplementation may be indicated to correct for insufficient amino acid intake in healthy individuals, and in specific physiological or pathophysiological situations**.** However, there is a concern to not supplement beyond the tolerable upper intake level (UL) by determining parameters of no-observed-adverse-effect level (NOAEL) or lowest-observed-adverse-effect level (LOAEL) for each amino acid. Since the NOAEL and LOAEL values are at least one order of magnitude different when comparing the values obtained in rats and humans, the aim of this review is to evaluate to what extent the amino acid UL measured in the rat model, when referenced to the dietary usual consumption (UC) and dietary requirement (RQ) for indispensable amino acids, may be used as an approximation of the UL in humans. This review then compares the ratios of the NOAEL or LOAEL over UC and RQ in the rat model with the same ratios calculated in humans for the nine amino acids (arginine, serine, glycine, histidine, leucine, lysine, methionine, phenylalanine, and tryptophan) for which this comparison can be done. From the calculations made, it appears that for these 9 amino acids, the calculated ratios for rats and humans, although rather different for several amino acids, remains for all of them in the same order of magnitude. For tryptophan, tyrosine, and valine, the ratios calculated in rats are markedly different according to the sex of animals, raising the view that it may be also the case in humans.

## Introduction

Amino acid supplementation may be indicated in healthy individuals to correct for deficiency of one or several amino acids in the dietary protein consumed by humans. Amino acid supplementation may also be indicated in specific physiological or pathophysiological situations. Clinical and experimental studies have shown for instance that supplementation with specific amino acids or their metabolites may contribute to limit muscle wasting in vulnerable patient groups (Martinez-Arnau et al. 2020; Wandrag et al. 2015); and supplementation with specific amino acids in rats with chemically induced colitis has been shown to favorably accelerate the process of intestinal mucosal healing after an episode of mucosal inflammation (Liu et al. 2013).

Adequate intake of amino acids from dietary protein can be defined as the need, in appropriate amounts, of all amino acids required for protein synthesis/renewal and for utilization of amino acids in other metabolic pathways. Alternate pathways include energy production and synthesis of various compounds with biological activities in terms of metabolism and physiological functions. The need for these latter compounds represents generally a minor proportion of the amino acid utilization. However, this is not always the case. Glutathione synthesis, for instance, has been reported to represent a relatively large part of cysteine utilization in the body during infection and intestinal inflammation (Malmezat et al. 2000; Rémond et al. 2011). Among the 20 amino acids present in dietary proteins, 9 of them (isoleucine, leucine, valine, lysine, methionine, phenylalanine, threonine, tryptophan, and histidine) are considered as indispensable as they have to be provided in the diet to meet the body’s requirement (Gietzen and Rogers 2006). From a metabolic point of view, among the 9 indispensable amino acids, 2 amino acids (lysine and threonine) are considered as strictly indispensable, as they cannot be synthesized by the body, notably by reamination of their corresponding ketoacids (Reeds 2000). Among the 11 other amino acids, six of them (arginine, cysteine, proline, tyrosine, glutamine, and glycine) are classified as conditionally indispensable, because their biosynthesis capacity in the body may be insufficient compared to the requirement in specific physiological and pathophysiological situations (Fürst and Stehle 2004). For indispensable amino acids, the requirements have been estimated in humans, and, as performed in this review, these requirements can be usefully compared with the mean usual consumption from the diet and supplements referred as consumption from all sources.

There is a major concern to not supplement beyond the amino acid tolerable upper intake level (UL) by determining parameters including the no-observed-adverse-effect level (NOAEL) and the lowest-observed-adverse-effect level (LOAEL) for each amino acid. NOAEL, commonly defined as the highest experimental intake that is without measurable adverse effect, does not fully address the interpretation of risk based on toxicologically relevant effects, nor does it consider the progression of effects with respect to duration and/or dose (Dorato and Engelhardt 2005). The tolerable UL can be defined as the highest level of daily nutrient intake that is likely to pose no risks of adverse effects in healthy individuals in the general population (Kennedy and Meyers 2005; Pizzo and Benfenati 2016). The 2005 Food and Nutrition Board, Institute of Medicine (FNB/IOM) reports dietary reference intakes (DRI) for energy, carbohydrate, fiber, fat, fatty acids, cholesterol, protein, and amino acids, and recommends that UL should be preferentially related to the NOAEL values when available. Although the difficulty of this task should not be under-estimated, data obtained from experiments with animals, and from few clinical studies are helpful in an attempt to define such amino acid ULs, and to fix an upper limit of what can be safely consumed. The determination of such safe limits for each amino acid is dependent on the identification of relevant biological end-points. In humans, for obvious ethical reasons, there is a limitation of the parameters that can be measured, compared to animal models. By summarizing the data obtained from studies in animal models and clinical studies in volunteers, a large heterogeneity of experimental design appears regarding notably supplementation duration (short-, middle-, and long-term experiments), parameters measured, dietary context, and age and sex of individuals. From another point of view, based on the guiding principles for ethical use of laboratory animals for research, namely replacement, reduction and refinement (Baumans 2005), we consider that, due to a relatively large number of peer-reviewed rodent toxicological studies with amino acids, it is desirable to try to interpret these data in term of amino acid safety in human. In that way, we hope to stimulate both new clinically relevant analyses and to reduce future experiments with rodents.

Since the NOAEL and LOAEL values determined for some amino acids are different by at least one order of magnitude when comparing the values determined in rats and humans, the overall aim of this review is to evaluate to what extent the UL determined for some amino acids from the NOAEL and LOAEL measured in animal models in healthy situation are informative when compared to the amino acid supply in standard feed/diet, reported to the Usual Consumption (UC) and requirement (RQ) for tentative extrapolation from rats to humans, notably for the amino acids for which no UL have been proposed for humans. UC has been defined in volunteers in reference to the dietary consumption over 1 year, ideally on each day of the year, keeping in mind that the self-reported nature of the data may lead to bias compared to the true consumption (Illner et al. 2010). Food frequency questionnaire is often used to evaluate UC (Siddiqui et al. 2019). Indispensable amino acid requirements (RQ) have been determined as mg/kg BW/day in growing rats (Benevenga et al 1994) and humans. Since most of the experiments with animal models have been done using the rat model, we have focused on the data obtained with this animal model, and when possible, we have also taken into account the biological sex effect. This does not mean that other animal models, like the pig model, are not relevant for human extrapolation (Chalvon-Demersay et al. 2017). However, regarding the pig model, only few data are available regarding the determination of the upper safe limit for amino acid intake in supplements.

## Indispensable amino acids

### Histidine UL

L-histidine is incorporated into proteins and metabolized leading to the production of glutamate. Histidine is a component of the dipeptide carnosine, a compound notably involved in pH-buffering. In addition, this amino acid displays metal-ion chelation, antioxidant actions (Boldyrev et al. 2013), and anti-inflammatory effects (Andou et al. 2009). Finally, histidine is the precursor of the biogenic amine histamine.

In rats, the amount of histidine in the standard diet is 3.3 g histidine/kg diet (Reeves et al. 1993). If we consider that laboratory rats on standard normoproteic diet eat approximately 22 g food per day (Liu et al. 2014), this will represent 72.6 mg histidine per day, and thus, based on a rat body weight (BW) that is usually approximately 250 g at the time of biological parameter determination, we can calculate an UC equal to 290.4 mg histidine/kg BW/day (Table 1). The NOAEL for histidine, as determined using parameters of subchronic toxicity, is, respectively, for male and female rats 1631 and 1757 mg/kg BW/day, while the LOAEL is 3481 and 3592 mg/kg BW/day, respectively (Ikesaki et al. 1994). The NOAEL/UC ratios for male and female are thus 5.6 and 6.1, respectively. The LOAEL/UC ratios for male and female are 12.0 and 12.4, respectively (Table 1). Amount of each amino acid RQ is calculated with reported amino acid requirements for growth in rats (Benevenga et al. 1994). In this animal model, histidine RQ is 133.3 mg/kg/day (Table 2), thus below the UC. The NOAEL/RQ ratios for male and female are 12.2 and 13.2, respectively, while the LOAEL/RQ ratios for male and female are 26.1 and 26.9, respectively.

**Table 1:** Comparison between the no-observed-adverse-effect level (NOAEL) and lowest-observed-adverse effect level (LOAEL), over usual consumption (UC) ratios for indispensable amino acids in rats and humans

Indispensable Amino Acid	Rat studies						Human studies					
	UC	Sex	NOAEL	LOAEL	NOAEL/UC	LOAEL/UC	UC	Sex	NOAEL	LOAEL	NOAEL/UC	LOAEL/UC
	mg/kg BW/day						mg/kg BW/day					
L-Histidine	290.4	M	1631	3481	**5.6**	**12.0**	31.43	F	91.2	134.1	**2.9**	**4.3**
		F	1757	3592	**6.1**	**12.4**						
L-Isoleucine	519.2	M	1565	3008	3.0	5.8	50.71	NI	ND	ND	ND	ND
		F	1646	3702	3.2	7.1						
L-Leucine	959.2	M	3333	ND	**3.5**	ND	87.14	M	500.0	ND	**5.7**	ND
		F	3835	ND	**4.0**	ND						
L-Lysine	809.6	M	3357	ND	**4.1**	ND	75.29	M	85.7	107.1	**1.1**	1.4
		F	3986	ND	**4.9**	ND						
L-Methionine	290.4	M	630	1100	**2.2**	**3.8**	25.29	F/M (9/6)	46.3	91.0	**1.8**	**3.6**
		F	ND	ND	ND	ND						
L-Phenylalanine	545.6	M	1548	4903	2.8	9.0	48.57	M	169.3	ND	**3.5**	ND
		F	1555	4701	**2.9**	8.6						
L-Threonine	413.6	M	3267	ND	**7.9**	ND	43.14	NI	ND	ND	ND	ND
		F	3673	ND	8.9	ND						
L-Tryptophan	140.8	M	948	1735	**6.7**	12.3	13.00	F	98.4	ND	**7.6**	ND
		F	1956	3705	**13.9**	26.3						
L-Valine	616.0	M	3225	ND	5.2	ND	57.00	NI	ND	ND	ND	ND
		F	1853	3721	3.0	6.0						

UC is the Usual Consumption. In rats, UC is calculated, as explained in the text, according to the amount of each amino acid in standard diet designed to correspond to the requirement for rats (Reeves et al. 1993). NOAEL is the No Observed Adverse Effect Level. LOAEL is the Lowest Observed Adverse Effect Level. F and M indicate experiments performed with female and male rats, and women and men, respectively. ND means not determined. NI means not indicated. In humans, the values for UC originate from FNB/IOM 2005. The data indicated in bold allow rapid comparison of the ratios calculated in rats and humans when available

**Table 2:** Comparison between the no-observed-adverse-effect level (NOAEL) and lowest-observed-adverse effect level (LOAEL), over requirement (RQ) ratios for indispensable amino acids in rats and humans

Indispensable amino acid	Rat study						Human study					
	RQ	Sex	NOAEL	LOAEL	NOAEL/RQ	LOAEL/RQ	RQ	Sex	NOAEL	LOAEL	NOAEL/RQ	LOAEL/RQ
	mg/kg BW/day						mg/kg BW/day					
L-Histidine	133.3	M	1631	3481	**12.2**	**26.1**	11.0	F	91.2	134.1	**8.3**	**12.2**
		F	1757	3592	**13.2**	**26.9**						
L-Isoleucine	295.2	M	1565	3008	5.3	10.2	15.0	NI	ND	ND	ND	ND
		F	1646	3702	5.6	12.5						
L-Leucine	509.5	M	3333	ND	**6.5**	ND	34.0	M	500.0	ND	**14.7**	ND
		F	3835	ND	**7.5**	ND						
L-Lysine	438.1	M	3357	ND	**7.7**	ND	31.0	M	85.7	107.1	**2.8**	3.5
		F	3986	ND	**9.1**	ND						
L-Methionine	309.5	M	630	1100	**2.0**	**3.6**	10.1	F/M (9/6)	46.3	91.0	**4.6**	**9.0**
		F	ND	ND	ND	ND						
L-Phenylalanine	323.8	M	1548	4903	**4.8**	15.1	27.0*	M	169.3	ND	**6.3**	ND
		F	1555	4701	**4.8**	14.5						
L-Threonine	295.2	M	3267	ND	11.1	ND	16.0	NI	ND	ND	ND	ND
		F	3673	ND	12.4	ND						
L-Tryptophan	95.2	M	948	1735	**10.0**	18.2	4.0	F	98.4	ND	**24.6**	ND
		F	1956	3705	**20.5**	38.9						
L-Valine	352.4	M	3225	ND	9.2	ND	19.0	NI	ND	ND	ND	ND
		F	1853	3721	5.3	10.6						

RQ is the value of requirement. In rats, RQ is amino acid requirements for growing rats (Benevenga et al. 1994). NOAEL is the No Observed Adverse Effect Level. LOAEL is the Lowest Observed Adverse Effect Level. F and M indicate experiments performed with female and male rats, and women and men, respectively. ND means not determined. NI means not indicated. The data indicated in bold allow rapid comparison of the ratio calculated in rats and humans when available

*RQ of phenylalanine/tyrosine

In humans, the daily requirement for histidine is 11.0 mg/kg BW/day (Table 2), while the consumption from dietary sources and supplements (thus UC from all sources) average 2.20 g/day (FNB/IOM 2005), thus representing 31.43 mg/kg BW/day (Table 1). When volunteers were given histidine supplementation for 4 weeks, and when parameters including anthropometric analysis, determination of body composition, sleep patterns, dietary intake, and urine analysis were performed, no deleterious effects of histidine given as supplement up to 8 g/day were observed, this NOAEL value is corresponding to 91.2 mg/kg BW/day (Gheller et al. 2020) (Table 1). The NOAEL/UC ratio is 2.9, a value close to the one found in rats. As indicated in Table 1, the LOAEL for histidine has been determined to be 134.1 mg/kg BW/day (Gheller et al. 2020). The LOAEL/UC ratio is 4.3 (Table 1). The NOAEL/RQ and LOAEL/RQ ratios are 8.3 and 12.2, respectively (Table 2).

Then, for histidine, the NOAEL/UC ratios are not vastly different when comparing the data obtained in rats and humans, being equal to 5.8 and 2.9, respectively. However, for histidine, the LOAEL/UC ratios are much more different when comparing rats and humans, being equal to 12.2 and 4.3, respectively. The NOAEL/RQ ratios for histidine are not very different when comparing the data obtained in rats and humans, being equal to 12.7 and 8.3, respectively. The LOAEL/RQ ratios are rather different, but within the same order of magnitude, when comparing the data obtained in rats and humans, being equal to 26.5 and 12.2, respectively.

### Isoleucine UL

L-isoleucine, together with L-leucine and L-valine, are branched-chain amino acids. The branched-chain amino acids, in addition to being required for protein synthesis, can be transaminated in the presence of α-ketoglutarate allowing production of glutamate and the corresponding α-ketoacid (Duan et al. 2016). Each α-ketoacid can then undergo oxidative decarboxylation (Matsumoto et al. 2010, 2014). The products of decarboxylation can then undergo several steps of conversion allowing the synthesis of acetyl-CoA, acetoacetic acid, and succinyl-CoA.

In rats, the amount of isoleucine in standard diet is 5.9 g/kg diet (Reeves et al. 1993), and thus, based on the same calculation as used above represents an UC equal to 519.2 mg/kg BW/day (Table 1). RQ is 295.2 mg/kg/day (Table 2). Based on BW, food consumption, ophthalmic signs, protein and electrolytes in urine, hematological parameters, blood biochemistry, and histopathological analysis determined after 13 weeks of isoleucine supplementation, NOAEL of 1565 mg/kg BW/day in males and 1646 mg/kg BW/day in females have been proposed (Tsubuku et al. 2004a) (Table 1). Similarly, the LOAEL for isoleucine has been determined to be 3008 and 3702 mg/kg BW/day for male and female rats, respectively (Tsubuku et al. 2004a). The NOAEL/UC ratios for isoleucine are then equal to 3.0 and 3.2 for male and female rats, respectively. The LOAEL/UC ratios are equal to 5.8 and 7.1 for male and female rats, respectively (Table 1). The NOAEL/RQ ratios for male and female are 5.3 and 5.6, respectively. The LOAEL/RQ ratios for male and female are 10.2 and 12.5, respectively (Table 2). The calculated ratios for male and female are thus similar.

In humans, the daily isoleucine requirement is 15.0 mg/kg BW/day (Table 2), while the mean UC from all sources is 3.55 g/day (FNB/IOM 2005) thus representing 50.71 mg/kg BW/day (Table 1), a value largely above the requirement. No NOAEL or LOAEL have been determined in humans.

### Leucine UL

In rats, the amount of L-leucine in standard diet is 10.9 g/kg diet (Reeves et al. 1993), and thus represents an UC equal to 959.2 mg/kg BW/day. RQ is 509.5 mg/kg/day (Table 2). In female rats during gestation, supplementation of the diet for 11 weeks with an aqueous solution of leucine equal to 1000 mg/kg BW/day had little impact on BW, food intake, litter size, weight, and fetus characteristics (Mawatari et al. 2004). After consumption for 13 weeks of diet supplemented with increasing amounts of leucine, and based on parameters including BW, food consumption, organ weight, hematological parameters and blood biochemistry, the NOAEL for leucine was determined to be 3333 mg/kg BW/day for males and 3835 mg/kg BW/day for females (Tsubuku et al. 2004a). Thus, the NOAEL/UC ratios are equal to 3.5 and 4.0 for male and female rats, respectively (Table 1). The NOAEL/RQ ratios are equal to 6.5 and 7.5 for male and female rats, respectively (Table 2). Then, the calculated NOAEL/UC ratio for rats, whatever their sex, is equal to approximately 3.7. Similarly, the NOAEL/RQ ratios for male and female, whatever their sex, are equal to 7.0.

In humans, as indicated in Table 2, the estimated daily requirement of leucine is 34.0 mg/kg BW/day (FNB/IOM 2005). The mean amount of leucine provided from all sources is 6.10 g/day (FNB/IOM 2005), corresponding to an UC equal to 87.14 mg/kg BW/day (Table 1). This value is here again above the requirement. In adult men, based on parameters that include the upper maximal capacity to oxidize leucine, as well as concentrations of glucose, insulin, alanine aminotransferase, and ammonia in blood, it was found that leucine supplementation at doses above 550 mg/kg BW/day (39 g for a 70 kg BW volunteers), when given acutely, may be above the upper safe limit (Pencharz et al. 2012). In elderly men, the upper limit for leucine supplementation is similar to the value found in young men (Elango et al. 2016). The proposed total dietary leucine upper limit of safe intake for the elderly is 500.0 mg/kg BW/day (Cynober et al. 2016). This value represents 5.7-fold the UC (Table 1), a value not vastly different from the one calculated in rats. Based on the RQ equal to 34.0 mg/kg/day, the NOAEL/RQ ratio is 14.7 (Table 2).

Then, for leucine, the NOAEL/UC ratio is equal to 3.7 in rats, while in humans, the ratio is equal to 5.7. Thus, the ratios calculated for leucine are not vastly different between rats and humans. However, the NOAEL/RQ ratios average 7.0 and 14.7 for rats and humans respectively.

### Lysine UL

L-lysine, in addition to being incorporated into proteins, is a precursor of acetoacetyl-CoA, carnitine, and of the polyamine cadaverine.

In rats, the standard diet contains 9.2 g lysine/kg diet (Reeves et al. 1993), thus representing an UC equal to 809.6 mg/kg BW/day (Table 1). RQ is 438.1 mg/kg/day (Table 2). Supplementation with lysine in the diet given ad libitum at increasing doses for 13 weeks was performed in rats, and parameters including BW, diet consumption, water intake, ophthalmic signs, organ weights, renal function, and histological examination were used to determine the NOAEL for lysine. NOAEL for this amino acid was found to be 3357 mg/kg BW/day in male rats, and the value for females was 3986 mg/kg BW/day (Tsubuku et al. 2004b). The NOAEL/UC ratios are then 4.1 and 4.9 for male and female rats, respectively (Table 1). The NOAEL/RQ ratios for male and female are 7.7 and 9.1, respectively (Table 2). The NOAEL/UC and NOAEL/RQ ratios averaged 4.5 and 8.4, respectively, for rats whatever their sex.

In humans, as indicated in Table 2, the estimated daily RQ for leucine is 31.0 mg/kg BW/day (FNB/IOM 2005). The mean amount of lysine provided from all sources is 5.27 g/day (FNB/IOM 2005; Martin et al. 2004), and thus, the UC is 75.29 mg/kg BW/day (Table 1), a value markedly above the requirement. Supplementation with lysine can cause symptoms related to the gastrointestinal tract such as nausea, stomach ache, and diarrhea. These results led to propose a provisional NOAEL equal to 6000 mg/person/day (Hayamizu et al. 2019), thus corresponding to 85.7 mg/kg BW/day for an individual weighing 70 kg, while an LOAEL equal to 107.1 mg/kg BW/day has been determined (Hayamizu et al. 2019). Thus, the NOAEL and LOAEL/UC ratios are 1.1 and 1.4, respectively (Table 1). The NOAEL/RQ and LOAEL/RQ ratios are 2.8 and 3.5 (Table 2).

Thus, in the case of lysine, the NOAEL/UC ratio is much higher in rats (i.e., being equal to 4.5) than in humans (i.e., 1.1). The ratio of NOAEL/RQ for lysine is also higher for rats than for humans being equal to 8.4 and 2.8, respectively. In humans, the dose of lysine with no adverse effect is close to the UC.

### Methionine UL

L-methionine is considered one of the most deleterious amino acid when given in excess (Harper et al. 1970). Methionine metabolism in tissues, which is rather complex, is related to the synthesis of numerous compounds with biological activities. L-methionine, in addition to serving as a precursor for protein synthesis, is a substrate together with L-lysine for carnitine synthesis, a compound involved in the metabolism of fatty acids (Longo et al. 2016). Methionine can be converted in three steps into homocysteine. Importantly, S-adenosylmethionine that is produced at the first step is a methyl donor involved in more than 60 methylation reactions in the body including phosphatidylcholine and DNA synthesis, as well as histone methylation (Brosnan and Brosnan 2006). These two latter methylation processes represent an important component for the regulation of gene expression in cells (Etchegaray and Mostoslavsky 2016). Then, homocysteine can be converted back to methionine in the pathways involving vitamin B_12_, 5-methyl tetrahydrofolate (5-methyl THF) and betaine, or be converted to cysteine in a vitamin B_6_-dependent pathway. Cysteine is the precursor, together with glutamate and glycine, of the tripeptide glutathione (GSH) that is involved in the control of the intracellular redox status and of the intracellular concentrations of reactive oxygen and reactive nitrogen species (Kemp et al. 2008; Chakravarthi et al. 2006). Cysteine is also the precursor of taurine, a compound notably involved in bile acid conjugation after bile acid synthesis from cholesterol (Chen et al. 2012). Last but not least, cysteine by itself, or in conjunction with homocysteine, is a precursor for the synthesis of hydrogen sulfide (H_2_S) (Blachier et al. 2019). Hyperhomocysteinemia, that can be observed in volunteers after supplementation with methionine (Chao et al. 2000), has been associated with increased risk for atherosclerotic and atherothrombotic vascular diseases (Zylberstein et al. 2004). However, the causal link between hyperhomocysteinemia and risk of atherosclerotic disease remains controversial (Blachier et al. 2020). Indeed, it appears that lowering homocysteinemia by itself is not sufficient for decreasing the risk of cardiovascular disease progression in volunteers (Ebbing et al. 2008), and that reduced synthesis of other compounds related to methionine and cysteine metabolism, like phosphatidylcholine and hydrogen sulfide, is also presumably detrimental when considering the risk of atherosclerosis (van der Veen 2017; Mani et al. 2013).

In rats, the amount of methionine in standard diet is 3.3 g/kg diet (Reeves et al. 1993), thus representing an UC equal to 290.4 mg/kg BW/day (Table 1). RQ for methionine is 309.5 mg/kg/day (Table 2), thus close to UC. Based on parameters including food intake, body weight, white blood cell count, thymus atrophy, and histological abnormalities in the adrenal gland and testis, the NOAEL found for methionine added to the diet of male rats for 4 weeks is 630 mg/kg BW/day, thus not vastly different from the UC, while the LOAEL is 1100 mg/kg BW/day (Chin et al. 2015). Thus, the NOAEL for methionine in supplement/UC ratio is 2.2, and LOAEL for methionine in supplement/UC ratio can be approximated to 3.8 in rats (Table 1). Since RQ for methionine is 309.5 mg/kg/day, then the NOAEL/RQ and LOAEL/RQ ratios are 2.0 and 3.6, respectively (Table 2).

In humans, the estimated RQ for methionine plus cysteine is 15.0 mg/kg BW/day (FNB/IOM 2005), while the RQ for methionine in the presence of an excess of dietary cysteine is 10.1 mg/kg BW/day (Di Buono et al. 2001). The amount of methionine provided by all sources (UC) is approximately 1.77 g/day (FNB/IOM 2005; Martin et al. 2004), thus representing an UC equal to 25.29 mg/kg BW/day for an individual weighing 70 kg, a value above the requirement. Based on plasma homocysteine as the primary determinant, but also on numerous other biochemical blood variables, as well as on answers to questionnaires regarding quality of life and cognitive function, the NOAEL for methionine is 46.3 mg/kg BW/day, while the LOAEL is 91.0 mg/kg BW/day (Deutz et al. 2017). Thus, the NOAEL for methionine/UC ratio is 1.8, and LOAEL/UC ratio is 3.6, these ratios being not vastly different from the ratios determined in rats (Table 1). Considering the RQ for methionine in the presence of an excess of cysteine, the NOAEL/RQ and LOAEL/RQ ratios are 4.6 and 9.0, respectively (Table 2).

Then, regarding methionine, which is considered as one of the most deleterious amino acid when consumed in excess, we have calculated from the available data that both the LOAEL/UC and the NOAEL/UC ratios are close in rats and humans, while the NOAEL/RQ and the LOAEL/RQ ratios are higher for humans than for rats.

### Phenylalanine UL

L-phenylalanine is incorporated into proteins and is a precursor of tyrosine and acetoacetyl-CoA.

In rats, the amount of phenylalanine in standard diet is 6.2 g/kg diet (Reeves et al. 1993), thus representing an UC equal to 545.6 mg/kg BW/day (Table 1). RQ is 323.8 mg/kg/day (Table 2). Based on the parameters including BW and food consumption measurement, organ weight, ophthalmic observation, red blood cell count, and blood glucose concentration measured after 4 weeks of ad libitum consumption of diet supplemented with phenylalanine, the authors proposed an NOAEL equal to 1.5% (w/w) for this amino acid (Shibui et al. 2014). The NOAEL for phenylalanine then represents 1548 and 1555 mg/kg BW/day in male and female rats, respectively (Shibui et al. 2014), and the LOAEL represents, respectively, 4903 and 4701 mg/kg BW/day (Shibui et al. 2014). Thus, the NOAEL/UC ratios are 2.8 and 2.9 for male and female rats, respectively. The ratio of LOAEL over UC is 9.0 and 8.6 for male and female rats, respectively (Table 1). The NOAEL/RQ ratios for male and female rats are both equal to 4.8. The LOAEL/RQ ratios for male and female are 15.1 and 14.5, respectively (Table 2).

In humans, the RQ for aromatic amino acids (phenylalanine/tyrosine) is 27.0 mg/kg BW/day (Table 2), and the mean intake of phenylalanine from all sources is 3.40 g/day (FNB/IOM 2005), and thus corresponds to an UC equal to 48.57 mg/kg BW/day (Table 1). The NOAEL for phenylalanine is 169.3 mg/kg BW/day (Miura et al. 2021). Thus, the NOAEL/UC ratio is 3.5, these ratios being close to the ratios determined in rats (Table 1). The NOAEL/RQ is 6.3 for humans (Table 2).

### Threonine UL

L-threonine is used for protein synthesis and for the synthesis of glycine and acetyl-CoA. Threonine is abundant in some proteins, such as intestinal mucins, that protect the intestinal epithelium against the deleterious effects of some compounds present in the intestinal luminal content (Munasinghe et al. 2017). The amount of threonine in standard diet for rats is 4.7 g/kg diet (Reeves et al. 1993), and thus represents an UC equal to 413.6 mg/kg BW/day (Table 1). RQ is 295.2 mg/kg/day (Table 2). By measuring parameters like BW, food consumption, food efficiency, ophthalmic signs, hematological and blood biomarkers after 13 week supplementation with threonine, the NOAEL for this amino acid was determined to be 3267 and 3673 mg/kg BW/day in male and female rats, respectively (Aoki et al. 2014^a^). The NOAEL/UC ratios are 7.9 and 8.9 for male and female rats, respectively (Table 1). The NOAEL/RQ ratios for male and female are 11.1 and 12.4, respectively (Table 2).

In humans, the threonine requirement has been estimated to be 16.0 mg/kg BW/day (Table 2), and the mean amount of threonine from all sources is 3.02 g/day (FNB/IOM 2005), that corresponds to an UC equal to 43.14 mg/kg BW/day (Table 1). No NOAEL or LOAEL has been determined in humans.

### Tryptophan UL

L-tryptophan, in addition to its precursor role for protein synthesis, is also a precursor for numerous compounds with biological activities, like the neurotransmitters serotonin and tryptamine, the hormone melatonin, and the vitamin-like compounds niacin and nicotinic acid (Kaluzna-Czaplinska et al. 2019). Tryptophan catabolism also leads to the synthesis of kynurenine, which controls several neurotransmitter systems (Schwarcz and Stone 2017). Moreover, tryptophan is a precursor for acetyl-CoA synthesis.

In rats, the amount of tryptophan in standard diet is 1.6 g/kg diet (Reeves et al. 1993), and thus represents an UC equal to 140.8 mg/kg BW/day (Table 1). RQ is equal to 95.2 mg/kg BW/day (Table 2). Based on BW and food consumption as well as other parameters including organ weight, histopathological analysis, and blood glucose measured after 13 weeks supplementation, the NOAEL for the tryptophan is 948 mg/kg BW/day in males and 1956 mg/kg BW/day in females. The LOAEL is 1735 and 3705 mg/kg BW/day for male and female rats, respectively (Shibui et al. 2018). Thus, the NOAEL/UC ratios in male and female rats are 6.7 and 13.9, respectively. The LOAEL/UC ratios in male and female are 12.3 and 26.3, respectively (Table 1). The NOAEL/RQ ratios for male and female are 10.0 and 20.5, respectively. The LOAEL/RQ ratios for male and female are 18.2 and 38.9, respectively (Table 2). The reasons for such huge difference for NOAEL and LOAEL between male and female rats are not known, but may rely on differences in tryptophan metabolism according to the animal sex. Measurement of maximal tryptophan oxidation rate in males and females may represent a way to measure the intake above which risk of adverse effects appear (Moehn et al. 2012).

In humans, the tryptophan requirement is 4.0 mg/kg BW/day (FNB/IOB 2005) (Table 2). The mean amount of tryptophan provided from all sources is 0.91 g/day (FNB/IOM 2005), thus representing an UC equal to 13.0 mg/kg BW/day, a value greatly above the requirement (Table 1). In healthy adult women, based on parameters like food intake, BW, blood and urine biomarkers, amino acid concentration in blood and urine, and profile of mood states category, the dose of 5.0 g/day of tryptophan (thus 98.4 mg/kg BW/day) used in supplement for 3 weeks showed no adverse effects (Hiratsuka et al. 2013). Thus, the NOAEL/UC ratio in humans can be calculated to be 7.6, a value relatively close to the NOAEL/UC ratio found in male rats, but lower than the ratio calculated for female rats (Table 1). On another hand, the NOAEL/RQ ratio for women is 24.6, a value relatively close to the value of NOAEL/RQ ratio found in female rats (Table 2).

### Valine UL

In rats, the amount of valine in standard diet is 7.0 g/kg diet (Reeves et al. 1993), thus representing an UC equal to 616.0 mg/kg BW/day (Table 1). RQ is 352.4 mg/kg BW/day (Table 2). After 13 weeks of valine supplementation, measurement of different parameters including BW, dietary consumption, ophthalmic signs, urine analysis, hematological and blood biochemical analysis, organ weight, and histopathological observation, allowed to propose an NOAEL for valine equal to 3225 mg/kg BW/day for males, with value for females being 1853 mg/kg BW/day. In female rats, the LOAEL has been estimated to be 3721 mg/kg BW/day (Tsubuku et al. 2004a). Thus, the NOAEL/UC ratios are 5.2 for males and 3.0 for females. In a similar way, the LOAEL/UC ratio is 6.0 for females (Table 1). The NOAEL/RQ ratios for male and female are 9.2 and 5.3, respectively. The LOAEL/RQ ratio for female is 10.6 (Table 2).

In humans, the daily requirement for valine is 19.0 mg/kg BW/day (Table 2), while the consumption from all sources is on average 3.99 g/day (FNB/IOM 2005), that corresponds to an UC equal to 57.00 mg/kg BW/day (Table 1), a value once again markedly above the requirement. No NOAEL or LOAEL has been determined in humans.

## Conditionally indispensable and dispensable amino acids

### Alanine UL

L-alanine, in addition to being used for protein synthesis, is a main substrate for gluconeogenesis. Alanine can be transaminated with α-ketoglutarate allowing the synthesis of pyruvate and glutamate.

In rats, the standard diet supplies 3.3 g alanine/kg diet (Reeves et al. 1993), thus representing an UC equal to 290.4 mg/kg BW/day (Table 3). Alanine supplementation by gavage for 4 weeks with 2000 mg/kg BW/day in rats indicated that this dose of alanine is well tolerated by the animals (Aoki et al. 2014b). This conclusion was drawn from the measurement of several parameters including BW, food consumption, ophthalmologic signs, urinary and blood biochemistry, hematological parameters, organ weight, and from histopathological analysis. Then, the NOAEL/UC ratio can be calculated to be equal to 6.9 for both males and females.

**Table 3:** Comparison between the no-observed-adverse-effect level (NOAEL) and lowest-observed-adverse effect level (LOAEL), over usual consumption (UC) ratios for dispensable amino acids in rats, mice (L-glutamate), and humans

Dispensable amino acid	Rat study						Human study					
	UC	Sex	NOAEL	LOAEL	NOAEL/UC	LOAEL/UC	UC	Sex	NOAEL	LOAEL	NOAEL/UC	LOAEL/UC
	mg/kg BW/day						mg/kg BW/day					
L-Alanine	290.4	M	2000	ND	6.9	ND	52.00	NI	ND	ND	ND	ND
		F	2000	ND	6.9	ND						
L-Arginine	396.0	M	3318	ND	**8.4**	ND	59.71	M	277.5	ND	**4.6**	ND
		F	3879	ND	**9.8**	ND		F	304.0	ND	**5.1**	ND
L-Asparagine	ND	M	1537	3242	ND	ND	89.86	NI	ND	ND	ND	ND
		F	1708	3466	ND	ND						
L-Aspartic acid	704.0	M	697	1417	1.0	2.0	93.43	NI	ND	ND	ND	ND
		F	715	1470	1.0	2.1						
L-Cysteine	211.2	M	ND	500	ND	2.4	14.43	NI	ND	ND	ND	ND
		F	ND	ND	ND	ND						
L-Glutamate	ND	M	6000	ND	ND	ND	ND	NI	ND	ND	ND	ND
		F	7200	ND	ND	ND						
L-Glutamine	ND	M	833	1654	ND	ND	ND	M	175.0	ND	ND	ND
		F	964	1984	ND	ND						
Glycine	202.4	M	2000	ND	**9.9**	ND	45.86	F	129.0	ND	**2.8**	ND
		F	ND	ND	ND	ND						
L-Proline	1258.4	M	2773	ND	2.2	ND	74.43	NI	ND	ND	ND	ND
		F	3009	ND	2.4	ND						
L-Serine	589.6	M	2765	ND	**4.7**	ND	50.29	M	169.3	ND	**3.4**	ND
		F	2905	ND	**4.9**	ND						
L-Tyrosine	580.8	M	600	2000	1.0	3.4	39.86	NI	ND	ND	ND	ND
		F	200	600	0.3	1.0						

UC is the Usual Consumption. In rats, UC is calculated, as explained in the text, according to the amount of each amino acid in standard diet designed to correspond to the requirement for rats (Reeves et al. 1993). NOAEL is the No Observed Adverse Effect Level. LOAEL is the Lowest Observed Adverse Effect Level. F and M indicate experiments performed with female and male rats, and women and men, respectively. ND means not determined. NI means not indicated. In humans, the values for UC originate from FNB/IOM (2005). The data indicated in bold allow rapid comparison of the ratio calculated in rats and humans when available

In humans, the mean alanine uptake from all sources corresponds to 3.64 g/day (thus representing an UC equal to 52.00 mg/kg BW/day, FNB/IOM 2005). No NOAEL or LOAEL has been determined in humans.

### Arginine UL

L-arginine, in addition to its role as a precursor for protein synthesis, can be converted to other amino acids present in proteins (notably glutamate, proline) or not present in proteins (ornithine, a precursor for the polyamine synthesis, and citrulline). Arginine is also the precursor for urea, creatine, and nitric oxide (NO). Arginine is used in the hepatic urea cycle for ammonia disposal, while polyamines are involved in cell proliferation, differentiation, and apoptosis, as well as in protein and nucleic acid synthesis and structure (Pegg 2016). Creatine can be phosphorylated into phosphocreatine that is involved in skeletal muscle activity (Strumia et al. 2012). NO regulates numerous physiological functions, notably due to its potent vasodilating effect (Napoli and Ignarro 2009). Arginine is able to stimulate the secretion of numerous hormones including insulin (Blachier et al. 1989) and growth hormone (Andersen 2015).

In rats, the standard diet contains 4.5 g arginine/kg diet (Reeves et al. 1993), and this represents an UC equal to 396.0 mg/kg BW (Table 3). Based on parameters including BW, food consumption, organ weight, and histopathological signs, the NOAEL for arginine after 13-week arginine supplementation in rats was found to be 3318 mg/kg BW/day in males and 3879 mg/kg BW/day in females (Tsubuku et al. 2004c). Thus, the arginine NOAEL/UC ratios in rats are equal to 8.4 for male and 9.8 for female rats (Table 3).

In humans, the mean amount of arginine provided by the diet is 4.18 g/day (FNB/IOM 2005), and thus represents an UC equal to 59.71 mg/kg BW. In overweight or obese but otherwise healthy volunteers, the measurement of blood pressure, serum concentrations of amino acids, glucose, fatty acids and related metabolites, as well as the measurement of renal, hepatic, and metabolic parameters, indicated that supplementation with L-arginine at a dose of 30 g/day for 90 days can be considered as safe. The NOAEL for arginine in men and women is, respectively, 277.5 and 304.0 mg/kg BW/day (McNeal et al. 2018). Then, the NOAEL/UC ratios is 4.6 and 5.1, respectively for men and women (Table 3). Thus, the NOAEL/UC ratio for human whatever their sex is approximately 4.9. When considering the observed safe level (OSL) of arginine, a somewhat different parameter from the NOAEL and LOAEL, Shao and Hathcock propose to not go beyond an amount of 20 g arginine per day, thus representing 285.7 mg/kg BW/day for 70 kg BW individuals (Shao and Hathcock 2008). This value is similar to previous value of NOAEL, i.e., 290.7 mg/kg BW/day, whatever the biological sex.

Then, for arginine, the NOAEL/UC ratio in rats is equal to 9.1, while the NOAEL/UC ratio in humans is lower being equal to 4.9.

### Asparagine UL

L-asparagine is primarily incorporated into the proteins of the body, but is also converted to aspartate and ammonia.

A 90-day toxicity study was performed in male and female rats with increasing amounts of asparagine added to the diet by measuring several parameters including BW, organ weight, blood biochemistry, as well as histological observation (Yokohira et al. 2008). From the data obtained, the authors have proposed an NOAEL for asparagine equal to 1537 mg/kg BW/day for males, and 1708 mg/kg BW/day for females (Table 3). The LOAEL for asparagine is 3242 and 3466 mg/kg BW/day in male and female rats, respectively (Yokohira et al. 2008).

In humans, the diet supplies 7.4 g asparagine per 100 g alimentary proteins per day (FNB/IOM 2005). Then, based on a usual protein consumption of 85 g/day, we can estimate an UC for asparagine equal to 89.86 mg/kg BW/day. No NOAEL or LOAEL has been determined in humans.

### Aspartic acid UL

L-aspartic acid is used for protein synthesis, but is also transaminated with α-ketoglutarate into oxaloacetate and glutamate. Aspartate is used in the urea cycle for the conversion of citrulline into argininosuccinate. Aspartate is a precursor for pyrimidine synthesis. Finally, aspartate is an energy substrate for several cell phenotypes including the intestinal epithelial cells (Windmueller and Spaeth 1976).

In rats, the dietary supply of aspartic acid is 8.0 g/kg diet (Reeves et al. 1993), thus representing an UC equal to 704.0 mg/kg BW/day (Table 3). In this model, supplementation with increasing amounts of aspartate was performed for 90 days, and parameters including serum and urinary parameters, weight, and histological examination of the kidneys and salivary glands were determined (Tada et al. 2008). From the data obtained, an NOAEL of 697 mg/kg BW/day is proposed for males, while the proposed value for females is 715 mg/kg BW/day. The LOAEL for aspartic acid in male and female rats represent 1417 and 1470 mg/kg BW/day respectively (Tada et al. 2008). Then, the NOAEL/UC ratio is 1.0 for both male and female rats (Table 3). Thus, from this study, it appears that the NOAEL for aspartate is close to the UC. The LOAEL/UC ratios are 2.0 and 2.1 for male and female rats, respectively (Table 3).

In humans, the consumption of aspartic acid from the diet and supplements averages 6.54 g/day (FNB/IOM 2005), thus representing an UC equal to 93.43 mg/kg BW/day. No NOAEL or LOAEL has been determined in humans.

### Cysteine UL

L-cysteine metabolism is described briefly in the above paragraph concerning methionine. Usual cyst(e)ine (cysteine + cystine) supply for rats from diet has been estimated to be 2.4 g/kg diet (Reeves et al. 1993), and thus represents 211.2 mg/kg BW/day. Based on several parameters including renal injuries, focal erosion in the stomach mucosa, and reticulocyte counts recorded in male rats after 4 weeks supplementation given by gavage once a day, a cysteine LOAEL equal to 500 mg/kg BW/day was proposed, but no NOAEL was reported in this study (Shibui et al. 2017). From this study, we can calculate an LOAEL/UC ratio equal to 2.4 (Table 3).

In humans, the mean amount of cysteine provided by the diet is 1.01 g/day (FNB/IOM 2005), and thus represents an UC equal to 14.43 mg/kg BW/day. No NOAEL or LOAEL has been determined in humans.

### Glutamine and glutamate UL

L-glutamine is the amino acid with the highest plasma concentration, while L-glutamate concentration in blood is among the lowest (Blachier et al. 2009). Both amino acids are metabolized extensively in the intestinal epithelium during their transcellular journey from the luminal content to the bloodstream (Blachier et al. 1999), serving as fuels for the enterocytes. The circulating concentration of glutamine is maintained by endogenous synthesis in the skeletal muscles, lung, and adipose tissue (Altman et al. 2016). Glutamine is a precursor for glutamate (and ammonia), that is then used as a precursor for alanine, aspartate, citrulline, and proline synthesis (Blachier et al. 2009). Glutamine is also serving for nucleotide synthesis. In addition, glutamate is a precursor, together with cysteine and glycine, for the synthesis of glutathione, and can be converted in minor amounts to N-acetylglutamate, an allosteric regulator of carbamoyl synthetase 1, an enzyme involved notably in the urea cycle (Caldovic and Tuchman 2003). Glutamate is involved in several aspects of taste and gastrointestinal physiology (Vandenbeuch and Kinnamon 2016; Boutry et al. 2011; Kouzuki et al. 2019; Hayabuchi et al. 2020), and is well known to be an excitatory neurotransmitter in the central nervous system (Meldrum 2000), as well as a precursor for the inhibitory neurotransmitter gamma-aminobutyrate (Turner and Whittle 1983).

In rats and human, the alimentary intake of glutamine with and without glutamate has not been determined.

In rats, regarding dietary L-glutamine, based on parameters including ophthalmologic signs, histopathological analysis, urine biochemistry, and hematological parameters, after 13-week supplementation with glutamine in an ad libitum design, it was possible to determine (Table 3) a glutamine NOAEL that was estimated to be 833 mg/kg BW/day in males and 946 mg/kg BW/day in females (Tsubuku et al. 2004d). Regarding the LOAEL, the values have been determined to be 1654 and 1984 mg/kg BW/day for male and female rats, respectively (Tsubuku et al. 2004d). Regarding L-glutamate, the NOAEL has not been determined in rats, but has been estimated in mice to be equal to 6000 and 7200 mg/kg BW/day in males and females, respectively (Anantharaman 1979).

In humans, an observed safe level of 14 g glutamine/day has been proposed (Shao and Hathcock 2008) that corresponds to approximately 175.0 mg glutamine/kg BW/day.

### Glycine UL

Glycine is used for protein synthesis and for the synthesis of serine and glutathione, as well as for the synthesis of creatine, purines, and heme in hemoglobin. Glycine is the most abundant amino acid in collagens (Albaugh et al. 2017).

In rats, the dietary supply of glycine from standard diet is 2.3 g/kg diet (Reeves et al. 1993), thus representing an UC equal to 202.4 mg/kg BW/day (Table 3). Glycine was given in increasing amounts by daily gavage, and BW, food and water consumption, organ weight, hematological and histopathological parameters, daily urinary chloride excretion, as well as other urinary biochemical data, and blood biochemical parameters were determined (Shibui et al. 2013). From the data obtained, the authors have proposed for male rats a glycine NOAEL equal to 2000 mg/kg BW/day. Then, the calculated NOAEL/UC ratio for glycine is 9.9.

In humans, the mean intake of glycine from all sources averages 3.21 g/day (FNB/IOM 2005), representing an UC equal to 45.86 mg/kg BW/day. The NOAEL for glycine is 129.0 mg/kg BW/day (Inagawa et al. 2006), thus allowing to calculate an NOAEL/UC ratio equal to 2.8.

Thus, for glycine, the NOAEL/UC ratio is 9.9 in rats, while this ratio is much lower in humans being equal to 2.8.

### Proline UL

L-proline is used for protein synthesis and is a precursor for glutamate synthesis. Proline and hydroxyproline are abundant in collagens (Albaugh et al. 2017).

In rats, the dietary supply of proline from standard diet is 14.3 g/kg diet (Reeves et al. 1993), corresponding to an UC equal to 1258.4 mg/kg BW/day (Table 3). Male and female rats were fed for 90 days with diet supplemented with proline and parameters including BW, food and water intake, urine composition, hematological signs, and serum biochemistry were measured (Tada et al. 2010a). From the data obtained, the authors proposed a proline NOAEL that represents 2773 mg/kg BW/day for males and 3009 mg/kg BW for females. Thus, the NOAEL /UC ratios are 2.2 and 2.4 for male and female rats, respectively. The calculated NOAEL/UC ratio for proline in rats, whatever their sex, is equal to approximately 2.3.

In humans, the mean consumption of proline from dietary sources and supplements is 5.21 g/day (FNB/IOM 2005) and thus the UC is equivalent to 74.43 mg/kg BW/day. No NOAEL or LOAEL has been determined in humans.

### Serine UL

L-serine, in addition to being used for protein synthesis, is a precursor for synthesis of the amino acids cysteine and glycine, and for the synthesis of the sphingolipids sphingosine and sphingosine-1-phosphate, which are important regulators of several cell functions (Pyne et al. 2016). Serine can also be metabolized to pyruvate.

In rats, the serine supply from the usual standard diet is 6.7 g/kg diet (Reeves et al. 1993), representing an UC equal to 589.6 mg/kg BW/day (Table 3). Male and female rats receiving diets supplemented with increasing amounts of serine for 90 days, and for which different parameters including BW, food intake, water intake, urine analysis, serum biochemistry, hematological signs, and organ parameters were analyzed, were characterized by minor changes in the hematological signs, serum biochemistry, and organ weight. These changes were, however, not associated with pathological signs (Tada et al. 2010b). From these data, the authors proposed that a serine NOAEL equal to 2765 mg/kg BW/day in males and 2905 mg/kg BW/day in females. The value found for serine NOAEL in another rat study was similar representing 3000 mg/kg BW/day (Kaneko et al. 2009). This study was performed for 13 weeks and was based on BW, food consumption, urinalysis and hematological parameters, serum biochemistry, organ weight, and histological signs. Thus, the NOAEL/UC ratios are 4.7 and 4.9 for male and female rats, respectively (Table 3). Then, the NOAEL/UC ratio for serine in rats whatever their sex is approximately 4.8.

In humans, the mean consumption of serine from dietary sources and supplements is 3.52 g/day (FNB/IOM 2005), thus representing an UC equal to 50.29 mg/kg BW/day. The NOAEL for serine supplementation is 169.3 mg/kg BW/day (Miura et al. 2021). Then, the NOAEL/UC ratio for serine in volunteers is 3.4, a value close to the one found in rats.

### Tyrosine UL

L-tyrosine, in addition to being incorporated into proteins, is a precursor for the catecholamines adrenaline, noradrenaline, and dopamine (Fernstrom and Fernstrom 2007). Tyrosine is also a precursor of the pigment melanin and of the hormone thyroxine.

In rats, the supply from usual diet is 6.6 g/kg diet (Reeves et al. 1993), thus representing an UC equal to 580.8 mg/kg BW/day (Table 3). Based on anatomical and histological considerations regarding the stomach, kidney, and liver, as well as on urine and blood biochemical parameters, recorded after supplementation of increasing doses of tyrosine given by gavage for 13 weeks in rats, the NOAEL for tyrosine was considered to be 600 mg/kg BW/day in males, and 200 mg/kg BW/day in females (Shibui et al. 2016). According to that study, the NOAEL for tyrosine appears surprisingly close to the UC for males, and even lower for females. Thus, the NOAEL/UC ratios for tyrosine are 1.0 and 0.3 for male and female rats, respectively. Regarding the LOAEL, the values for male and female rats are 2000 and 600 mg/kg BW/day, respectively (Shiubui et al. 2016). Thus, the LOAEL/UC ratios for tyrosine are 3.4 and 1.0 for male and female rats, respectively (Table 3). The reasons for increased sensitivity to the deleterious effects of tyrosine in excess, in females compared to males, remain to be determined.

In humans, the mean consumption of tyrosine from all sources is 2.79 g/day (FNB/IOM 2005), thus representing an UC equal to 39.86 mg/kg BW/day. No NOAEL or LOAEL has been determined in humans.

## Conclusion and perspectives

The comparison between rats and humans for the NOAEL and LOAEL for each amino acid either indispensable or dispensable for which studies have been performed is presented in Tables 1 and 3. From this analysis, several conclusions can be drawn. Importantly, by simply comparing the NOAEL and LOAEL obtained in the rat model and in humans, it appears clearly as expected, that the values obtained in rats cannot be extrapolated directly to humans. This is due to the fact that the usual amino acid consumption in rats (expressed as mg amino acid per kg BW per day) is one order of magnitude higher than amino acid consumption in humans. This is related to the fact that protein consumption per kg BW is approximately tenfold higher in rats compared to humans (Dubuisson et al. 2010; Pasiakos et al. 2015).

Thus, it appears much more relevant to compare, as done in this review, the NOAEL and LOAEL/UC ratios together with NOAEL and LOAEL/RQ ratios between the rat model and humans. However, due to the limited number of studies, this can be done only for the 9 amino acids arginine, glycine, histidine, serine, leucine, lysine, methionine, phenylalanine, and tryptophan. Overall, from the calculations made, it appears that for these 9 amino acids, the calculated ratios for rats and humans, although much different for several amino acids, remain for all of them within the same order of magnitude. All these data should be interpreted taking into account that the UC of indispensable amino acids originating from diet and supplement in Western countries like the USA and France are largely above the requirement.

Apart from these 9 amino acids, such comparison between rats and humans cannot be done due to a lack of data for the other 11 amino acids (isoleucine, threonine, valine, alanine, asparagine, aspartic acid, cysteine, glutamate, glutamine, proline, and tyrosine). We can only relate to the rat studies indicating that, depending on which of the 11 amino acids were tested, different NOAEL/UC ratios can be calculated ranging from 0.7 for tyrosine to 8.4 and 9.1 for arginine and threonine, respectively (Tables 1 and 3). For indispensable amino acids, different NOAEL/RQ ratios can be calculated for rats ranging from 4.8 for phenylalanine to 11.8 for threonine (Table 2). This suggests that in the rat model, some amino acids consumed above the RQ or UC are well tolerated according to the parameters measured. At the opposite, for amino acids characterized by NOAEL close to the RQ or UC, deleterious effects are likely to be observed at doses not greatly above the RQ or UC. This raises the view that a same situation may prevail in humans, as suggested by the few available data.

Using the rat model generally allows to have access to more physiological, metabolic, and functional parameters than the ones that can be obtained from clinical studies with volunteers. Then, it is tempting to propose that the ratios calculated in the rat model represent a rough but useful approximation of the corresponding ratios in humans, notably for future clinical trials with amino acids that have not been yet studied in volunteers. Second, it appears that for some amino acids (tryptophan, valine, and tyrosine, see Tables 1 and 2), the NOAEL or LOAEL measured in rats are much different based on the sex of the animals, raising the possibility that it might be also the case for humans.
